# Spheres Derived from Lung Adenocarcinoma Pleural Effusions: Molecular Characterization and Tumor Engraftment

**DOI:** 10.1371/journal.pone.0021320

**Published:** 2011-07-18

**Authors:** Rita Mancini, Enrico Giarnieri, Claudia De Vitis, Donatella Malanga, Giuseppe Roscilli, Alessia Noto, Emanuele Marra, Carmelo Laudanna, Pietro Zoppoli, Pasquale De Luca, Andrea Affuso, Luigi Ruco, Arianna Di Napoli, Giuseppe Mesiti, Luigi Aurisicchio, Alberto Ricci, Salvatore Mariotta, Lara Pisani, Claudio Andreetti, Giuseppe Viglietto, Erino A. Rendina, Maria Rosaria Giovagnoli, Gennaro Ciliberto

**Affiliations:** 1 Department of Clinical and Molecular Medicine, University of Rome “La Sapienza” S. Andrea Hospital, Rome, Italy; 2 Biogem s.c.a.r.l., Ariano Irpino (AV), Italy; 3 Department of Experimental and Clinical Medicine, University of Catanzaro “Magna Graecia”, Catanzaro, Italy; 4 Department of Thoracic Surgery, University of Rome “La Sapienza”, Sant'Andrea Hospital, Rome, Italy; 5 Charles River Laboratories, Research Model and Services, Lecco, Italy; 6 Department of Biological and Bioenvironment Study, University of Sannio, Benevento, Italy; 7 Department of Biology, College of Science and Technology, Temple University, Philadelphia, Pennsylvania, United States of America; The University of Texas MD Anderson Cancer Center, United States of America

## Abstract

Malignant pleural effusions (MPEs) could represent an excellent source to culture a wide variety of cancer cells from different donors. In this study, we set up culture conditions for cancer cells deriving from MPEs of several patients affected by the most frequent form of lung cancer, namely the subset of non small cell lung cancers (NSCLC) classified as Lung Adenocarcinomas (AdenoCa) which account for approximately 40% of lung cancer cases. AdenoCa malignant pleural effusions gave rise to in vitro cultures both in adherent and/or in spheroid conditions in almost all cases analyzed. We characterized in greater detail two samples which showed the most efficient propagation in vitro. In these samples we also compared gene profiles of spheroid *vs* adherent cultures and identified a set of differentially expressed genes. Finally we achieved efficient tumor engraftment in recipient NOD/SCID mice, also upon inoculation of small number of cells, thus suggesting indirectly the presence of tumor initiating cells.

## Introduction

In recent years methodological approaches to study cancer have been deeply influenced by two major emerging concepts: a) cancer is not a single disease but rather a heterogeneous composition of multiple diseases, where we are just starting to distinguish and classify subtypes characterized by distinct genetic and epigenetic changes and by the activation of different signal transduction pathways and transcriptional programs [1]–[3]; b) the growing importance of the cancer stem cell theory [4]–[9]. This claims that cancer is originated by a population of cells with stem cell like properties (CSCs), whose distinctive feature is their extensive capacity of self renewal, and which sustain cancer growth through the production of a pool of transiently amplifying expanded progenitor cells and of terminally differentiated progenies [10]–[13]. The putative CSCs are themselves mostly quiescent for proliferation, are resistant to chemotherapeutic agents and therefore are thought to be responsible for disease relapse and for the emergence of resistance to therapies [14]. These two concepts have led investigators to shift their focus from well established cell lines adapted by several years in culture to primary tumors because of the realization that a) cell lines do not reproduce the natural heterogeneity of tumors and b) they have undergone a series genetic and epigenetic changes following years of adaptation in culture and do not maintain the hierarchical organization typical of the original tumor. Hence from here increasing efforts to establish and characterize banks of primary tumors [15], [16].

Although evidence has been provided that this approach can be applied to obtain interesting correlations with clinical outcome in solid cancers [17], its medium-high throughput implementation suffers from several limitations. Putative CSCs are generally present in low amounts in a given tumor tissue and proliferate slowly; in addition very often the amount of available tumor tissue obtained by surgery is too small. Therefore the proportion of tumors from which it is possible to set up primary cultures of bona fide putative CSCs is generally low and only in a subset of cases these cells are capable of engraftment in vivo [18]–[20]. This has led to the development of methods to enrich for subpopulations of cells with increased tumorigenic potential using either surface markers such as CD133 [9] or CD44 [21]; functional activities such as alhedyde dehydrogenase (ALDH) staining or Hoechst 33342 exclusion [22], [23]; or also in vitro culture systems in suspension as spheroids in well defined medium conditions [24], followed by the demonstration that engraftment of small amounts of cells in recipient mice is able to regenerate a complete tumor tissue resembling histologically and functionally the originating human tumor. Although there is no general consensus about the best combination of surface markers to use, it is common belief that an enrichment step is helpful for the efficient establishment of tumors in vivo [25], [20].

Lung cancer is the leading cause of cancer mortality in the world. Current therapy is relatively ineffective and survival rate at 5-years remains only 15% for advanced disease [26]. Malignancy is one of the main causes of pleural effusions, and 90% of malignant pleural effusions (MPEs) are due to metastatic disease [27], [28]. Carcinomas of any organ can metastasise to the pleura, but the most frequent ones are lung carcinomas. Very recently a limited number of studies have demonstrated that MPEs could be an excellent source of tumor initiating cells, because they are capable to efficiently propagate in vitro and in vivo, and to reproduce the natural heterogeneity of tumors. One of these studies analyzed malignant pleural effusions from breast cancer, the other pleural effusions from a small set of heterogenous lung cancers. Interestingly, Grimshaw et al. demonstrated that the ability to induce tumors appeared to correlate with the ability to produce large spheres, but not with a particular expression of known surface markers [20], [29], [30]. We collected a set of samples and assessed their ability to give rise to adherent *vs* non adherent spheroid cultures without applying prior enrichment for surface markers. By analyzing the global pattern of gene expression we also started to derive a signature distinctive of spheroid *vs* adherent AdenoCa cultures. Finally we demonstrated efficient engraftment in vivo in mice and the establishment of tumors which reproduce the histological features of the primary human tumors.

## Materials and Methods

### Sample collection, processing and cultures

We selected 15 cases of malignant pleural effusion obtained from patients with lung adenocarcinoma at the Department of Thoracic Surgery, University of Rome La Sapienza, Sant'Andrea Hospital. After thoracentesis, all specimens were received as fresh effusion, usually within one hour following collection, with volumes ranging from 40 to 1,500 ml. After centrifugation for 5 min at 1,500 rpm, the supernatant was decanted and the sediment resuspended in 30 ml of CytoLyte Solution (Cytyc Corp., Massachusetts, USA). The specimen was then recentrifuged for 10 min at 1,500 rpm and the sediment resuspended in 20 ml of PreservCyt solution (Cytyc Corp.). A monolayer slide was made using the Thin Prep 2000 Processor (Cytyc Corp.). From each specimen, 5–10 slides were prepared. Of these, 1 or 2 were stained using the Papanicolaou method, and viewed independently to confirm the diagnosis and presence of cells suitable for evaluation. Tumor type was determined according to cytomorphology parameters: solitary and clusters of medium to large sized cells, hyperchromatic and eccentrical nuclei, conspicuous nucleoli and variable cytoplasmatic vacuolization. Malignant pleural fluid cells were also submitted to immunocytochemistry in order to confirm Adenocarcinoma cell origin. Diagnoses were considered ‘true positives’ if they were inagreement with the consensus cytological diagnosis.

Experiments were approved by the Sant' Andrea Hospital Ethics Committee 2010 (504/10). All patients agreed to participate to the study and signed an informed consent form.

To partially purify tumor cells, cells from Malignant Pleural Effusion (MPE) were subjected to discontinuous density gradient centrifugation as follows. MPE, obtained aseptically in heparinized (10 U/ml) bottles and processed within a reasonable time (4 hr), were centrifuged at 300 g for 10 min, at 4°C and cell pellet was resuspended in 30 ml of cold 1% BSA/2 mM EDTA/PBS. Cells were counted in a haemocytometer by Trypan Blue exclusion dye (Sigma-Aldrich) and then cell suspension was layered on modified Histopaque-1077 (Sigma-Aldrich) modified gradient in 50 ml conical tube (Falcon). Histopaque-1077 solution of a density of about 1,065 gr/ml was obtained by diluting it with Phosphate Buffered Saline pH 7,2. Discontinuous gradient was then centrifuged at 800 g, for 30 minutes, at room temperature. During the centrifugation step the cells were separated according to their different buoyant densities: the denser fluid components such as erythrocytes and some leucocytes migrate into the lower phase through the bottom of the tube while the less dense cell fraction, including tumor cells, are enriched at the interphase. After centrifugation, cells contained in the upper gradient and in the pellet were collected and washed once with 1%BSA/2 mM EDTA/PBS. Cells from the upper gradient were then plated in “Sphere Medium” at a concentration of 100'000 cells/ml in non-tissue culture treated plates (Falcon) or Ultralow binding plates (Corning). Half volume medium was replaced once a week while growth factors (bFGF and EGF) were added every other day. The pellet from density gradient centrifugation, which contains leucocytes, erythrocytes and clumps of tumor cells, was subjected to hypotonic lysis to remove erythrocytes, washed once in 10% FCS-RPMI (Invitrogen) and then plated in tissue culture treated Petri dishes (Falcon).

To determine their self renewal ability, sphere propagation assay were performed: adherent lung cancer cells were mechanically dissociated and plated in non-adherent culture to form P0 spheres. After 6–12 days individual spheres were formed, dissociated with Accumax and serial dilution was used to replate an average of 1 cell per well in 96-well plates to generate P1 spheres. This procedure were repeated several up to 3–4 times.

### ALDH activity assay

Cells growing in adherent or spheroid conditions, were collected, dispersed by Accumax (Innovative Cell Technologies, Inc. San Diego, CA) treatment, and 0.5–1×10^6^ cells analyzed for ALDH1 activity using ALDEFLUOR kit (Stem Cell Technologies) following manufacturer instruction. Briefly, cells were suspended in ALDEFLUOR assay buffer containing the fluorescent ALDH1 substrate, BODIPY-aminoacetaldehyde (BAAA), and incubated for 45 min at 37°C. The assay buffer contains also a transport inhibitor, to prevent efflux of the BAAA from the cells. BAAA passively diffuse into live cells and then is converted by intracellular ALDH1 into a negatively charged product BODIPY-aminoacetate, which is retained inside cells, labeling them with a bright fluorescent signal. After a washing step, the brightly fluorescent ALDH1-expressing cells (ALDH1^br^) were detected in the green fluorescence channel (FL1; 520–540 nm) of a FACSCalibur instrument (BD Biosciences). A sample of cells were stained as above with the addition of a specific ALDH1 inhibitor, diethylaminobenzaldehyde (DEAB) (Sigma), to serve as a negative control for each experiment. Because only cells with an intact cellular membrane could retain the ALDH1 reaction product, only viable ALDH1br cells were identified. Cells incubated with BAAA and DEAB were used to establish the background signal and to define the ALDH1^br^ region. Incubation of cells with the substrate in the absence of DEAB induced a shift in BAAA fluorescence defining the ALDH1^br^ population. Data were analyzed by FCSExpress.

### Microarray Analysis

Single strand biotinylated cDNA was generated as follows: 100 ng of total RNA were subjected to two cycles of cDNA synthesis with the Ambion WT expression Kit (Applera). The first cycle – first strand synthesis is performed using an engineered set of random primers that exclude rRNA-matching sequences and include the T7 promoter sequences. After second-strand synthesis, the resulting cDNA is in vitro transcribed with the T7 RNA polymerase to generate a cRNA. This cRNA is subjected to a second cycle – first strand synthesis in the presence of dUTP in a fixed ratio relative to dTTP. Single strand cDNA is then purified and fragmented with a mixture of uracil DNA glycosylase and apurinic/apirimidinic endonuclease 1 (Affymetrix) in correspondence of incorporated dUTPs. DNA fragments are then terminally labeled by terminal deoxynucleotidyl transferase (Affymetrix) with biotin. The biotinylated DNA was hybridized to the Human Genechip Gene 1ST Arrays (Affymetrix), containing almost 29000 genes selected from *H. sapiens* genome databases RefSeq, ENSEMBL and GenBank. Chips were washed and scanned on the Affymetrix Complete GeneChip Instrument System, generating digitized image data (DAT) files.

Data files were analyzed with AGCC (Affymetrix, Santa Clara, CA) producing CEL files. Robust multichip average (RMA) normalization and data analysis were performed using GeneSpring 11.0.2 (Agilent Technologies, Santa Clara, CA). Time analysis among the different stages of the same culture was performed using CAGED (Cluster Analysis of Gene Expression Dynamics) which identifies the most probable set of clusters given a set of time series [31]. The culture identified as PE e/10 has 6 time points, therefore, according to CAGED's recommendations for short time series, we used the Polynomial Model approach (Model Order = 3, Prior Precision 1, Gamma Value = 0, Bayes Factor = 1, Distance = Euclidean). We obtained 12 clusters, however only one showed a significant variation over time. This cluster, containing 69 probesets, was functionally annotated by Gene Ontology (GO) for Biological Process. Enriched GO terms (i.e. terms with a significantly higher than expected number of associated genes) were filtered (*p*≤1*10^−2^) by the Hypergeometric test and corrected using False Discovery Rate (FDR). For the analysis of gene expression profiles in adherent cultures compared to sphere cultures, differentially expressed (DE) probe sets were filtered for fold change ≥1.5. Statistical analysis was performed using the Welch *t*-test, with *p*≤0.05. DEGs were functionally characterized using a proprietary software, Ingenuity Pathway Analysis (IPA) from Ingenuity Systems® to identify possible enriched molecular networks and canonical pathways. From the 29000 genes on the chip we obtained 1494 differentially expressed probe sets for the patient PE d/10 and 171 differentially expressed probe sets for the patient PE e/10. At this point we generated the intersection list of the two samples composed by 82 probe set. As a further filtering step, we generated a list of probes with similar behavior in the two samples by considering just those genes with a FC lower than 1.5 between the two adherent cultures. In this way we produced a curated list of 54 DE probe sets. The accession for experimental submission is MIAMExpress database number E-MEXP-3025.

### Quantitative RT-PCR (pRT-PCR)

Using Primer3 software analysis available on the web, we selected primer sequences to optimally amplify target for qRT-PCR assay. To avoid possible amplification of contaminating genomic DNA primers were designed so that each PCR product covered at least one intron. All the primers used have been synthesized by Sigma. The expression of selected genes was examined by real-time reverse transcription-PCR (RT-PCR) using SYBR Green Detection Chemistry (Applied Biosystem). One micrograms of total RNA extracted from each of the samples were used to make complementary DNA (cDNA). Twenty nanograms of cDNA were used as template for qPCR amplification in presence of 0,2 µM specific primers, in a total volume of 25 µl. PCR reactions without cDNA samples were used as negative controls. Each reaction was done in triplicate and repeated at least twice to verify the results. SDS system software (Applied Biosystems, Foster City, CA) was used to convert the fluorescent data into cycle threshold (Ct) measurements and the relative amount of specific transcript was calculated by the comparative cycle threshold method given by [32]–[33]. All values of RNA accumulation of the specific genes were normalized to the signal of GAPDH and HPRT in each sample and plotted for comparison of the relative amounts of the transcripts. Statistical analyses were performed using the SPSS® computer program package (SPSS for Windows, version 11.5). Frequency tables were analysed using t- Student's correlation coefficient. The statistical significance was set at *P*<0.05.

### Generation of subcutaneous and orthotopic lung cancer xenografts into NOD/SCID mice

Two-month-old male NOD/SCID mice (Charles River, Margate, Kent, UK) were used. Animals were anesthetized with ketamine and injected with 10^4^ cells subcutaneously and with 2×10^3^ cells intra lung. Mice were housed in a highly controlled microbiological environment to guarantee SPF conditions. They were maintained in IVC cages, under constant conditions of temperature (22±2°C), humidity (55%±10 UR) and light/dark cycle of 12/12 hours. The animals had free access to irradiated standard diet and water.

Cells obtained by detachment from PE d/10 and PE e/10 adherent or spheres cultures dissociated to obtain single cell suspension, were diluted in 1× PBS plus 50% matrigel before subcutaneous injection. Mice were daily monitored to avoid any sign of suffering and weekly to check for the appearance of subcutaneous tumor or weight loss due to potential tumor growth. When subcutaneous tumors volume reached at least 700 mm^3^ in size, the tumor mass was excised, collected and submitted to histological analysis. In a subset of cases tumors were minced into small pieces and transplantated into a second recipient NOD/SCID mouse. In absence of additional signs of pathology groups of mice were euthanized every two months in order to check for the presence of lung cancer metastasis. At animal sacrifice, a complete necropsy procedure was performed. Cervical, thoracic, abdominal, and pelvic organs were extracted en bloc. We assessed the development of local tumor at the injection site, measured its two largest diameters, and recorded all macroscopic tumor deposits or abnormalities in any organ, especially those in which lung cancer foci were expected (pleura, lymph nodes, liver, and brain). The whole block of organs was fixed with buffered formalin for 48 h, except for the lung. The lungs were excised and the tumor removed, weighed, fixed with 10% formalin in PBS. Two general pathologists analyzed histopathologically the tumor at the injection site. All the experimental procedures were approved by the Ethical Committee for the Animal Use (CESA) of the IRGS and where communicated to the Italian Ministry of Health.

### Immunocytochemistry and NOD/SCID mice immunohistochemistry

PE d/10 and PE e/10 were submitted to cytospins using 100000 cells per spot, centrifuged at 800 rpm for 3 minutes. The slides were allowed to dry over night and fixed with Acetone or Formaldheyde 4% for intracitoplasmatic antigens or membrane antigens, respectively. Cytospins were washed in PBS tween20 0.05%. Blocking reagent followed by primary antibody and then biotinylated anti-mouse secondary antibody were added. The primary antibody used was an anti-CK7 (DAKO Cytomatic), anti-BerEP4 (DAKO Cytomatic) anti-Vimentin (Novocastra) and ALDH 1a1 (Epitomics). After a further step of washing in PBS tween 20 0.05%, slides were treated with avidin-biotin complex for 30 min using DAKO Cytomatic LSAB+Syste-HRP detection system according to the manufacturers instructions. Staining was performed with 3,3′-diaminobenzidin and counterstaining with HE. The primary antibodies were omitted and replaced with preimmune serum in the negative control. Positive controls were adopted based on the manufacturer's instructions.

Sections 3 µm thick from Xenograft mice tumor were cut onto silanized glass slides and air-dried overnight at room temperature. Sections were dewaxed in xylene and rehydrated through graded alcohol. After peroxidase inhibition, the sections were immersed in citrate buffer (pH6.0) (Zymed, San Francisco, CA, USA) and irradiated twice in a microwave oven (800W) at full power for 15 min. After, the slides were incubated with the following antibodies: anti-CK7 (DAKO Cytomatic), anti-BerEP4 (DAKO Cytomatic) anti-Vimentin (Novocastra). After a further step of washing in PBS, slides were treated with avidin-biotin complex for 30 min using Dako Cytomation LSAB+Syste-HRP detection system according to the manufacturers instructions. Staining was performed with 3,3′-diaminobenzidin. and counterstaining with HE. At least 100 cells per view were counted. Cases were considered positive if at least 10% of cells were strongly stained for the antibody. The primary antibodies were omitted and replaced with preimmune serum in the negative control. Positive controls were adopted based on the manufacturer's instructions.

## Results

### Lung adenocarcinoma cells isolated from malignant pleural effusions give rise to primary cultures

Malignant pleural effusions were collected by thoracentesis as described in the Materials and Methods section and as previously reported [34]. All samples were subjected to cytological and immunocytochemistry analysis (see Materials and Methods). We decided to establish primary cultures only of samples diagnosed cytologically as lung adenocarcinomas.

All samples, grew in RPMI medium in the presence of 10% FBS although, as expected, a great variability from sample to sample was observed with respect to doubling time and duration of cell culture (not shown). In order to avoid long periods of adaptations in cell culture that may lead to the selection of subpopulation of cells with particular growth advantages all samples were banked in liquid nitrogen at passage 3 (p3), on average one month following MPE collection. All further studies were conducted with p4 to p6 cells, i.e. cells in the initial passages after thawing, unless otherwise specified.

**Figure 1:**
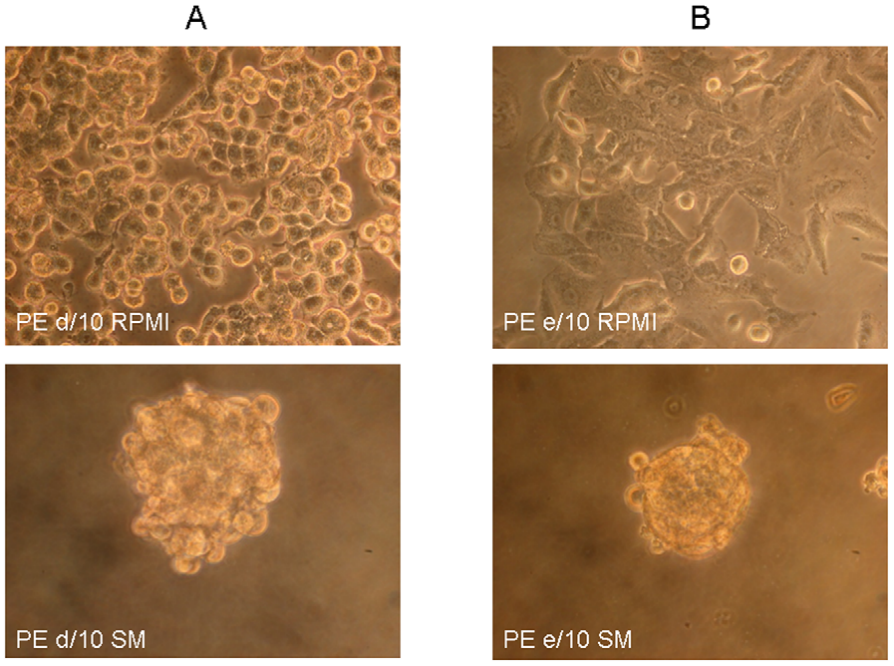
a: In the upper panel a sample picture of PE d/10 and PE e/10 cell culture grown in adherence in RPMI medium in presence of serum; b: in the lower panel the same primary cultures grown in non-adherent conditions supplemented with growth factors (bFGF and EGF) (200×).

p4 cells all re-grew in adherence. When they were transferred into sphere forming medium (SM), i.e. DMEM/F12 medium supplemented with EGF and FGF, the majority of samples, i.e. 11 out of 15 (**Table 1**), produced spheres propagating through some passages, although, also in this case, significant variability was observed among samples with respect to the sphere forming unit ability and sphere size. Some examples are shown in **Figure 1**: sample PE d/10 grows in adherence as cells with epithelial shape forming a cobblestone-like monolayer and as large irregular spheroids in sphere medium (**Figure 1a**). In contrast, in the case of sample PE e/10, cells grew in large clusters in part also made of cells with elongated and fibroblastoid shape in RPMI condition, but form tinier and very compact spheres in sphere medium condition (**Figure 1b**).

**Table 1:** Clinicopathological features of pleural effusion cases.

Case n°	Sex	Age	Primary site	T	N	M	P-Stage	Survival	RPMI	Sphere Medium
PE a/10	F	77	RUL	4	3	1a	IV	4	yes	no
PE b/10	M	81	RLL	4	2	1b	IV	1	yes	yes
PE c/10	F	85	RLL	4	3	1a	IV	11	yes	No
PE d/10	M	64	RIL	4	3	1a	IV	0	yes	Yes
PE e/10	F	52	LLL	4	1	1a	IV	0	yes	Yes
PE f/10	M	76	RUL	4	2	1a	IV	14	yes	No
PE g/10	F	63	LLL	4	3	1b	IV	4	yes	Yes
PE h/10	M	78	RML	4	2	1a	IV	6	yes	Yes
PE i/10	M	66	RML	4	3	1a	IV	4	yes	Yes
PE l/10	M	77	LLL	4	2	1a	IV	6	yes	Yes
PE lm10	M	57	LLL	4	2	1a	IV	3	yes	Yes
PE n/10	F	51	LUL	4	3	1a	IV	0	yes	No
PE o/10	M	56	RLL	4	1	1a	IV	11	yes	Yes
PE p/10	M	77	RUL	4	2	1a	IV	2	yes	Yes
PE q/10	M	63	RUL	4	2	1a	IV	26	yes	Yes

Patient characteristics and culture performances of adenocarcinoma cells from pleural effusions in MPE, RPMI and SFM medium conditions.

In order to demonstrate the presence in primary cell line PE d/10 and PE e/10 of CSCs in our spheroid cultures we set up self renewal assays (see Materials and Methods section). Primary spheres were dissociated with Accumax and single cells seeded in 96-well plates. After 6–12 days we observed the formation of individual spheres (**Figure S1**). This procedure was repeated successfully at least 2 times showing, however, a gradual decrease in the frequency of sphere formation up to passages 3–4.

### AdenoCa spheroids from MPEs overexpress stem cell markers

**Figure 2:**
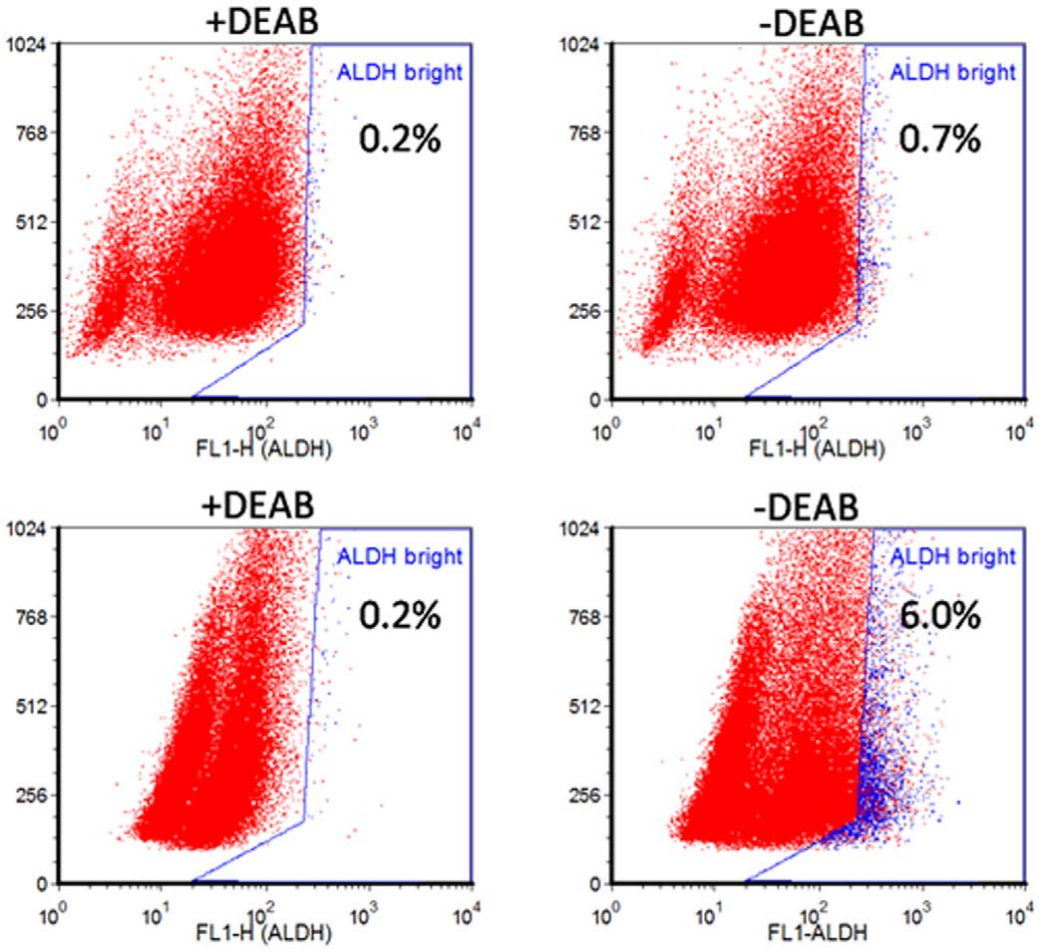
Showed the presence of PE e/10 percentage of ALDH^br^ cells which are increased upon culturing in spheroid conditions, providing information about the presence of putative CSC in MPE primary cultures.

Cancer Stem Cells (CSC) are single tumor cells which are thought to be capable to regenerate a tumor or a metastasis. The identification of CSC remains challenging and several CSC markers have been proposed. Among several, ALDH1 expression and its enzymatic activity seems to better correlate presence of CSC and aggressiveness of lung tumors [35]. Isolated ALDH1-positive cells from stable cell lines showed features of CSC and overexpression of ALDH1 has been correlated with poor prognosis for patients with early-stage NSCLC [22]. Primary cultures obtained from MPEs were characterized for the presence of cells with ALDH1 activity by FACS analysis; both in adherent and spheroid culture. Results (**Figure 2** **and** **Table 2**) show that in the majority of samples analyzed the percentage of ALDH^br^ cells increased upon culturing in spheroid conditions, providing information about the presence of putative CSC in MPE primary cultures.

**Table 2:** FACS analysis of ALDH activity.

MPE cultures	Adherent +DEAB	−DEAB	Floating +DEAB	−DEAB
**PE d/10**	**0.2%**	**1.1%**	**0.2%**	**3.3%**
**PE e/10**	**0.2%**	**0.7%**	**0.2%**	**6.0%**
**PE g/10**	**0.18%**	**5.2%**	**0.2%**	**5.1%**
**PE h/10**	**0.2%**	**2.1%**	**0.2%**	**7.5%**
**PE i/10**	**0.21%**	**1.5%**	**0.18%**	**4.8%**
**PE l/10**	**0.2%**	**1.7%**	**0.2%**	**5.3%**

Primary cultures grown in adherent or sphere conditions have been analyzed by FACS for ALDH activity using ALDEFLUOR assay. All samples, in adherent or sphere conditions, were incubated with or without ALDH inhibitor, DEAB, to determine baseline fluorescence.

**Figure 3:**
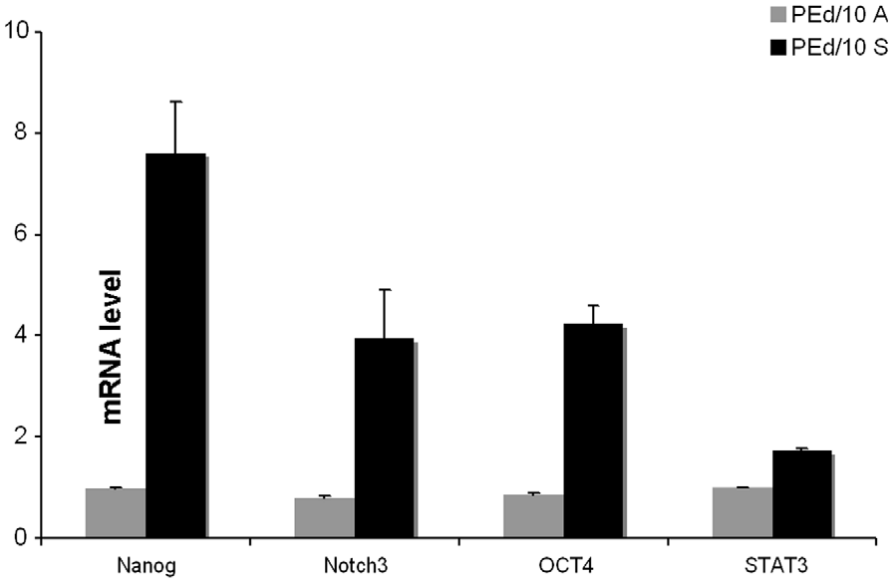
Real -time RT-PCR data. Stem cell markers in adesion and sphere cells. The data are presented as mean +/−SD of three independent experiments showing p<0.05 statistical analysis.

Moreover, the expression level of stem cell markers connected to self-renewal and stemness cabability was evaluated. To this purpose, we examined Oct-4, Nanog, Notch3 and Stat3 at level of transcription in RNA extracted from adherent *vs* shperoids of sample PE d/10. The amounts of the indicated transcripts, analyzed by real-time RT-PCR, were significantly increased in cell grown as spheres compared with those grown in adherence (**Figure 3**).

### A small subset of genes is differentially co-regulated in sphere *vs* adherent cells

**Figure 4:**
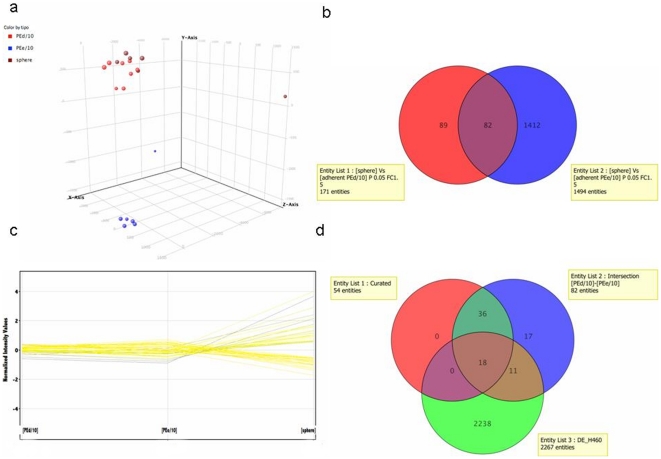
Bioinformatic analysis. a) PCA analysis on adherent culture PE d/10 (red), PE e/10 adherent culture (blue) and sphere culture (brown); b) Venn Diagram of the two adherent cultures showing 82 probesets in the Intersection; c) Gene expression profiles of the Curated list; d) Venn Diagram of the Curated list, Intersection list and DE probeset list of H460 cells.

**Figure 5:**
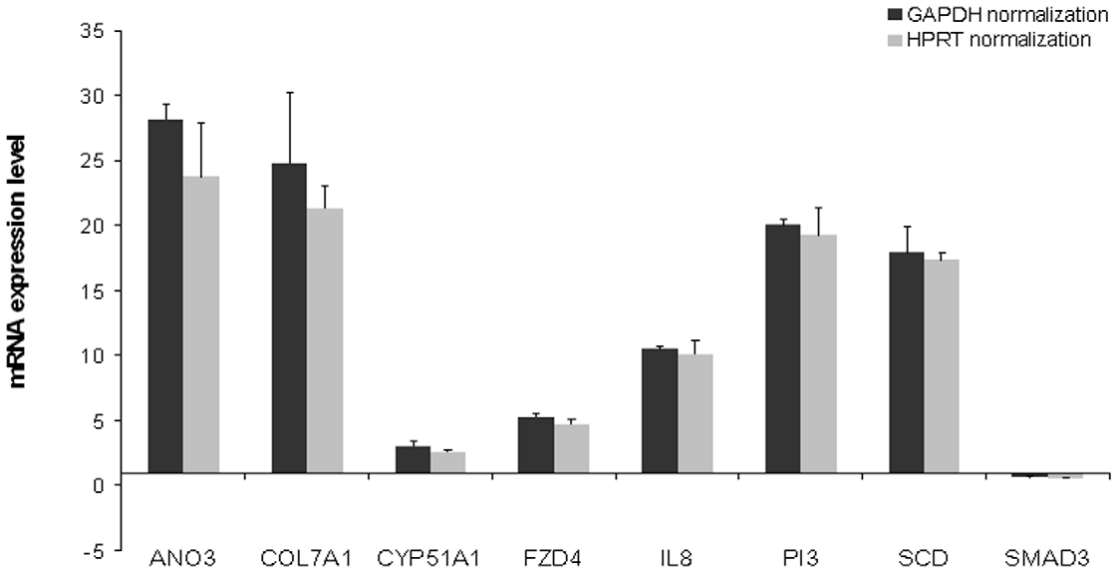
Results of qPCR analysis of 8 selected genes from the 18 DE gene list, confirming the fidelity of the array analysis (p<0.05). Expression Profile for all of the selected genes, in normalizations with two different housekeeping genes (GAPDH/HPRT).

In the previous sections we have shown that lung AdenoCa MPEs give rise with efficiency to cell cultures which are able to grow in adherent conditions and as spheroids. For the rest of our study we focussed our attention on two samples, i.e. PE d/10 and PE e/10 that were among the most efficient in *in vitro* propagation. Morphological and immunocytochemistry examination of these cultures over passages (p4 to p 10) did not reveal any substantial change (not shown). In order to better evaluate possible changes taking place over time in culture, RNA samples was extracted from adherent cultures of sample PE d/10 at passages 6, 8, 10, 13, 14 and 16 and subjected to expression profiling. We utilized CAGED an algorithm based on Bayesian Clustering by Dynamics (see *Methods section*), which performs Bayesian clustering on temporal gene expression data to search for the most probable set of clusters given the available data. The majority of gene clusters showed a virtually flat behaviour over time, thus confirming an overall stability of gene expression (not shown). Only one small cluster made by 69 probesets showed a moderately raising expression profile over time. (**Table S1**). When this cluster was functionally annotated by Gene Ontology (GO) for Biological Process (*p*≤1*10^−2^) we obtained 9 Biological Process GO categories enriched in the PE d/10. Interestingly the GO categories enriched in the PE d/10 are related to “DNA conformation changes” as reported in the supplementary (**Table S2**). Furthermore, we performed genome-wide microarray analysis to ask if it is possible to identify a set of genes that are differentially regulated between sphere cultures and adherent cells. We compared gene expression profiles of adherent vs sphere cultures of RNA extratcted at p6 (**Figure 4a**). Differentially expressed (DE) probe sets were filtered for fold change >1.5 statistical significance (*p*-value 0.05) resulting in 1494 DE probe sets for the PE d/10 adherent culture and 171 DE probe sets for the PE e/10. We then calculated the intersection list with a Venn Diagram (**Figure 4b**) to obtain a common DE probe sets of 82 genes in adherent *vs* spheres between the two patients (**Table S3**). To discard genes whose expression doesn't vary significantly between the two adherent cultures, this DE probe set was again filtered for fold change, this time looking for genes with a negligible variation (fold change value lower than 1.5) between the two adherent cultures (**Figure 4c**). In this way, from the initial 82 intersection DE gene list, we obtained a curated list of only 54 DE genes (**Table S4**). A bibliographic annotation on curated genes was performed using Ingenuity Pathway Analysis (IPA), as described in Materials and Methods. The most significant IPA Canonical Pathways are shown in **Figure S3**. We finally compared the previous 82 and 54 DE gene lists obtained from MPE primary cultures with unpublished profiling data obtained comparing adherent *vs* sphere cultures of the stable lung cancer AdenoCa cell line NCI-H460. The results, schematically represented in the Venn Diagram (**Figure 4d**), show an overlap of 29 DE genes which appear co-regulated (up-or down-regulated) in sphere *vs* adherent cultures of MPEs and NCI-H460 (**Table 3**). If only the curated gene list is considered, the overlap with NCI-H460 spheres *vs* adherent is reduced to only 18 genes. In order to start validating these results we carried out qPCR analysis of 8 selected genes from the 18 DE gene list (**Figure 5**). qPCR results confirmed the fidelity of the array analysis. Remarkably we obtained more consistent fold changes in RNA levels compared with the Array Expression Profile for all of the selected genes, in normalizations with two different housekeeping genes (GAPDH/HPRT). Therefore, the qPCR results confirm the accuracy of the Array analysis in determining differential gene expression profile of the cell growth in two different conditions, RPMI and SM.

**Table 3:** Sphere *vs* adherent cultures co-regulation.

Transcripts Cluster Id	Genesymbol	NCI-H460 (Sphere/Adherent) Gene Expression	MPE Gene Expression
7908459	CFH	UP	UP
8086985	COL7A1	UP	UP
8051583	CYP1B1	UP	UP
8140864	CYP51A1	UP	UP
7950885	FZD4	UP	UP
8095680	IL8	UP	UP
8040695	KCNK3	UP	UP
7985317	KIAA1199	UP	UP
7961365	MANSC1	UP	UP
8003332	MVD	UP	UP
8062927	PI3	UP	UP
7920244	S100A8	UP	UP
7905571	S100A9	UP	UP
8098195	SC4MOL	UP	UP
7929816	SCD	UP	UP
7941148	TM7SF2	UP	UP
7939024	TMEM16C	UP	UP
8028652	ZFP36	UP	UP
7994582	SULT1A3	UP	UP
7948667	AHNAK	DOWN	DOWN
8070632	CBS	DOWN	DOWN
7953291	CD9	DOWN	DOWN
7978706	FOXA1	DOWN	DOWN
7977854	JUB	DOWN	DOWN
8017019	MTMR4	DOWN	DOWN
7984364	SMAD3	DOWN	DOWN
8056545	STK39	DOWN	DOWN
8093950	S100P	UP	DOWN
7899615	SERINC2	UP	DOWN

Overlap of 29 DE genes which appear co-regulated (up-or down-regulated) in sphere *vs* adherent cultures of MPEs and NCI-H460.

### Pleural effusion adherent and spheroids cell derived are more efficient than cultures in the establishment of tumor growth in vivo in NOD/SCID mice

The best way to assess the presence of tumor initiating cells in a lung tumor cell culture sample is the demonstration of the ability to form tumors when implanted in vivo by transthoracic or subcutaneous puncture in NOD/SCID mice. For this reason we carried out engraftment studies with cells derived from PE d/10 and PE e/10 at passage p6. We injected a constant number of cells, namely 10^4^ for subcutaneous (s.c.) and 2×10^3^ for orthotopic injections respectively. Results indicate that both adherent and spheroids cultures engrafted very efficiently s.c. A compendium of data obtained upon s.c. implantation is reported in Table 4. Almost all injected mice developed large tumors (**Figure S2**) with a variable latency time of about 10–16 weeks (**Table 4**). We achieved also serial transplantation of one tumor from PE d/10 and one from PE e/10 (**Table 5**). Interestingly, secondary tumors appeared after a much shorter latency time, thus suggesting an increase in the number of tumor initiating cells after the first passage in mice. A more limited number of orthotopic injections were performed; in this case we observed that only spheroids were able to grow in the lung parenchyma (not shown).

**Table 4:** Characterization of generated tumors.

	No. of tumor cells inoculated	No. of subcutaneous tumors	Tumor volume (mm3)	Tumor Weight (gr)	Latency (weeks)
**PEe/10 adherent**	1×10^4^	5/6	1571.2 Ds 1013,3	20.7	12
**PEd/10 adherent**	1×10^4^	4/6	1786.2 Ds 980,5	29.3	14
**PEe/10 spheroid**	1×10^4^	13/16	1817.2 Ds 851,4	21.2	12
**PEd/10 spheroid**	1×10^4^	6/6	2538.1 Ds 1617,7	29.2	10

Table shows the number of inoculated cell and tumor developed in subcute with their characteristic of volume, weight and latency.

**Table 5:** Characterization of tumors after transplantation.

	Number of subcute tumors	Transplantation	Immunohistochemistry	Tumor volume(mm3)	Tumor weight (gr)	Latency (Weeks)
**PE e/10**	3	Subcutaneous	Identical to parental tumors	1021.5	23.1	6
**PE d/10**	3	Subcutaneous	Identical to parental tumors	971.7	22.4	5

Table shows numbers of tumors transplanted, site of transplantation, immunohistochemistry patterns, volume, weight and latency.

**Figure 6:**
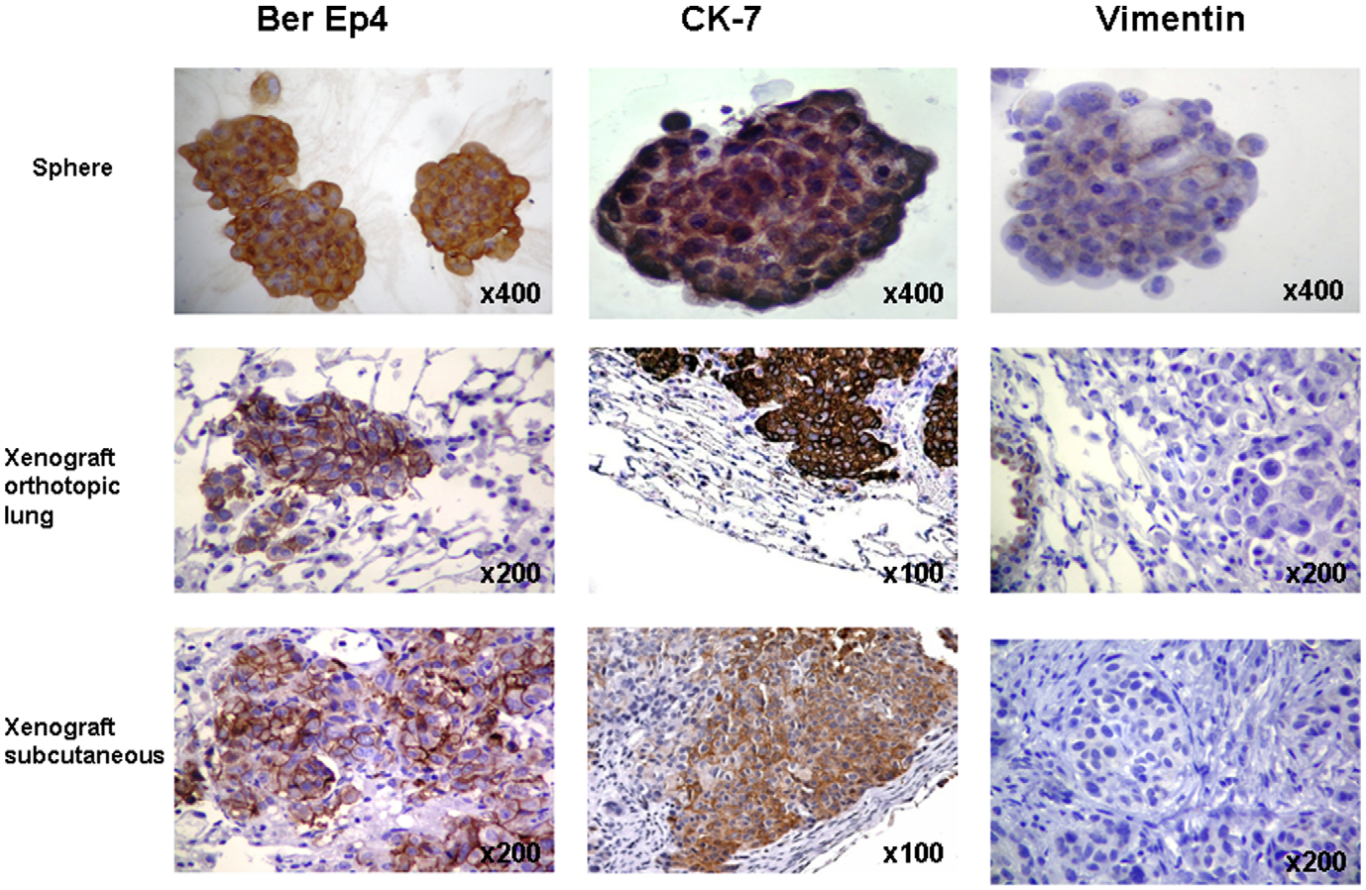
Immunophenotipic characteristics of human adenocarcinoma spheres and matched tumors generated in NOD SCID mice. Immunostaining for Ber-Ep4, CK 7 and Vimentin showed Ber-Ep and CK 7 protein expression in both spheres and xenografts; in contrast, a weak Vimentin expression was observed in cytological specimens only.

**Figure 7:**
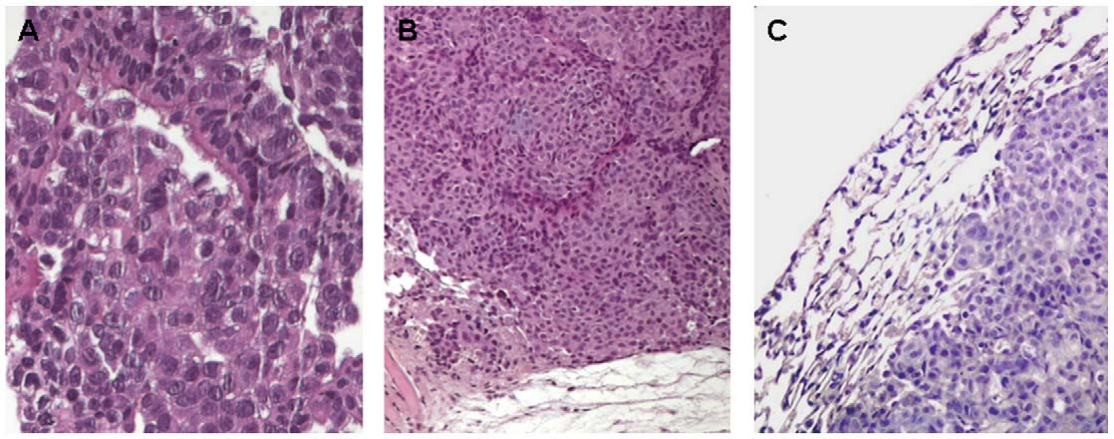
Histological features of lung adenocarcinoma xenografts. Representative histological images of H&E stained slides of primary lung tumor (a, original magnification ×200) and corresponding subcutaneous (b, original magnification ×100) and orthotopic (c, original magnification ×200) grafts. Adenocarcinomas reproduced in NOD SCID mice showed the same morphologic pattern of the primary tumor characterized by pleomorfic neoplastic cells arranged in solid nests.

**Figure 8:**
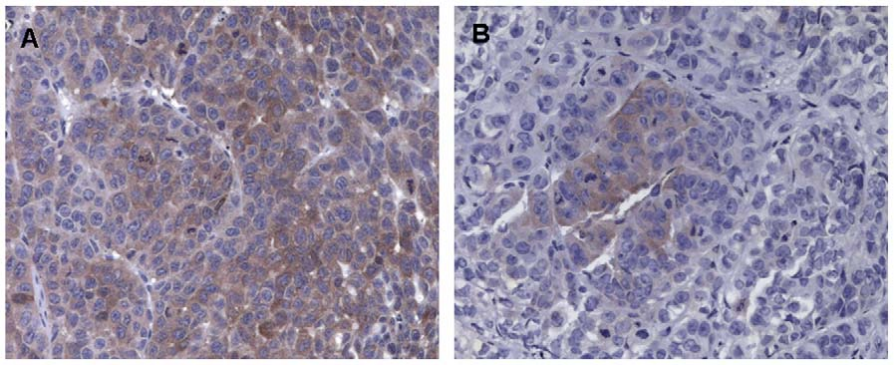
Immunohistochemistry for ALDH in subcutaneous tumor xenografts. A. Sphere implants in NOD/SCID mice showed citoplasmic staining for ALDH. B. Focal expression of ALDH was detected in adherent cells implants (original magnification ×200).

All implanted mice were euthanized after tumor development. Histological evaluation of all organs was then carried out which revealed adenocarcinoma lesions only in lung and subcutaneous tissues. Adenocarcinoma reproduced in NOD SCID mice showed pathologic features very similar to those seen in original human tumors; both xenografts and primary tumors were characterized by the presence of pleomorphic tumor cells with a predominantly solid pattern of growth (**Figure 6**). Immunohistochemical evaluation of CK7, BerEp4 and Vimentin markers expression was carried out on all xenograft lesions and on the spheroid cells before injection (**Figure 7**). All spheres showed strong expression of CK7 and BerEp4, and a weak staining for Vimentin. Subcutaneous and lung xenografts showed CK7 and BerEp4 immunoreactivity in all cases; none showed Vimentin expression. A final intriguing observation was that, in line with what observed in vitro, tumors developing upon adherent culture injections maintained in vivo a lower percentage of ALDH+ cells than their counterparts obtained upon spheroid injections (**Figure 8**).

## Discussion

The present study confirms and expands recent observations showing that Malignant Pleural Effusions (MPEs) obtained from lung cancer patients are a source of cancer initiating cells and may constitute an excellent model to study inter-and intra-tumoral heterogeneity [29]. Differently from the prior study reported in literature, we have decided to focus on samples derived from the most common form of NSCLC, namely Lung AdenoCa, and to assess their propagation in vitro using two different culture conditions. The first interesting observation is that MPE cell cultures maintain viability and are capable of propagation for some passages with high efficiency. As expected, morphology, duplication time, and length of duration in culture differs significantly from sample to sample, but the main observation is that a high proportion of samples is able to grow both as adherent and as spheroid cultures and can be banked for further studies.

We have decided to focus our attention on the two samples, PE d/10 and PE e/10 that were most efficient in propagation in vitro and have started to ask the following questions: a) how is the global pattern of gene expression changing over time in culture?; b) are spheroid cultures enriched in markers commonly associated with stemness and do they differ from adherent cultures in their gene expression programs?; c) are lung AdenoCa cultures from MPEs capable of efficient engraftment in vivo and, if so, can we observe differences between adherent *vs* spheroids?

In response to the first question, we carried out morphological and genome wide gene expression profiling using the Affimetrix platform in sample PE e/10 over serial passages. Although we are aware of the limitations given by the small set of samples analyzed, the conclusion that can be drawn is that the overall gene expression pattern in sample PE e/10 remains stable over time. Interestingly, however, a small cluster of genes enriched for genes involved DNA packaging and maintenance undergoes a statistically significant and gradual increase from passage 6 to 16. This indirectly reinforce the concept that long term culture of primary cells may lead to the occurrence of epigenetic changes. We are currently investigating if the same trend is observed in other samples of our collection.

In response to the second question, microarray analysis of samples PE d/10 and PE e/10 shows that they differ substantially in their pattern of gene expression (**Figure 4a**). This finding underscores the high degree of inter-patient heterogeneity of tumors from the same histotype. We believe that the difference observed is due to a different complement of oncogenes mutations and/or signalling pathways deregulations in the two samples. Indeed, although we have not conducted a thorough mutational analysis we have observed that while sample PE d/10 carries activating mutations of the Ras oncogene, sample PE e/10 does not possess a mutated Ras, but shows a prominent upregulation of several members of the ErbB receptor family and the constitutive activation of downstream Akt pathway (unpublished).

Since some studies have reported a higher efficiency of tumor engraftment of spheroid *v*s adherent cultures [36], we have started to look for genes that are differentially regulated. To this purpose we conducted two types of analysis. In first instance we studied the expression of a well defined set of known markers such as ALDH, Oct4, Nanog, STAT3 and Notch3 by FACS staining or RT-PCR and found, as expected, that spheroid cultures overexpress these genes. Secondly we compared by microarray analysis the global pattern of gene expression of adherent *vs* spheres and intersected data obtained from the two selected lung AdenoCa MPEs with those obtained by microarray analysis of RNA extracted from adherent *vs* sheroids of the NCI-H460 cell line. This led to the identification of a small set of 18 genes which show a common trend for up- or down-regulation in lung tumor spheres *vs* adherent cultures. A preliminary validation was performed successfully on a subset of 8 genes using RT-PCR. At this stage a complete validation of the entire set of 18 genes by RNA and protein analysis on the same and additional MPE samples is necessary to increase our confidence on the small signature we have identified. It is important to point out that some of the genes present in our small lung spheroid signature have been recently reported to be involved directly or indirectly in lung tumorigenesis. This is the case for example of FZD4 and SCD-1. FZD4 is one of the frizzled receptors responsible for signal transduction of Wnt/β-catenin pathway. Indeed Wnt/β-catenin signalling has been shown to potentiate cancer stem cell behaviour in lung cancer A549 cells [37]. With respect to SCD1, an enzyme responsible for the synthesis of monounsaturated fatty acids, there is mounting evidence that lipid perturbations are a common feature of cancer and cancer cells display a high rate of fatty acid synthesis propelled by excel substrates supplied by active glycolysis. Indeed, Scaglia et al have recently shown that reduction of SCD1 expression in lung cancer cells significantly delays tumor formation and growth rate in mouse xenografts [38]. Therefore, our profiling studies provides a short list of genes as promising candidates for studies directed to assess their functional contribution to spheroid formation and in vivo tumorigenicity. Albeit preliminary, we have indeed observed that knocking down expression of these two genes significantly impairs spheroid formation in vitro (AN, CDV and RM, unpublished observations).

In response to the last question we observed a high efficiency of tumor engraftment with p6 cells from the two selected samples when implanted into NOD/SCID mice. To our opinion this represents an outstanding rate of uptake, also in the light of our use of recipient NOD/SCID mice, which are known not to provide the most permissive environment for CSC transplantation as compared to NOD/SCID/IL2Rγ null mice [39]. Furthermore in two out of two cases tested we obtained secondary tumors, which also showed a much shorter latency time than the respective primary tumors (**Table 5**). Importantly, the tumor histology and the immunohistochemistry carried out with a limited number of markers indicates that tumors developing in mice closely resemble the original primary human tumor, albeit with some differences. This demonstrates that tumor cells grown in culture have not undergone significant deviation.

It is somehow surprising that within the same sample (PEd/10 or PE e/10) the rate of engraftment was similar when comparing spheroids *vs* adherent cultures. This is in apparent contrast with the observation that, at least in our initial survey of known markers commonly associated with stemness, these were significantly enriched in spheroids. Indeed, in the case of ALDH expression, we observed that tumors obtained upon inoculation of spheroids maintained a higher staining for ALDH than tumors obtained after inoculation of adherent cells. It is possible, therefore, that in order to detect more subtle differences between spheroids and adherent cultures in their uptake efficiency it will be necessary to conduct in vivo limiting dilution assays. This will deserve further analysis as well as a thorough understanding of the preliminary finding that only spheroids but not adherent cultures gave rise to tumors when injected orthotopically.

In conclusion we have shown here that MPEs are an excellent source of tumor initiating cells in lung AdenoCa. Bulk cultures can be utilized to identify genes linked with sphere formation in vitro and enhanced tumor propagation in vivo.

## Supporting Information

Figure S1 Sphere assays, self renewal ability of primary lung cancer cell.
(TIF)

Figure S2 NOD/SCID mice subcutaneous tumor growth.
(TIF)

Figure S3 IPA Canonical Pathways.
(TIF)

Table S1 Enriched GO terms and filtered by the Hypergeometric test and corrected using False Discovery Rate (FDR).
(TIF)

Table S2 Cluster, containing 69 probesets, was functionally annotated by Gene Ontology (GO) for Biological Process.
(TIF)

Table S3 69 probesets expression profile over time.
(TIF)

Table S4 Differential expressed probe sets of 82 genes in adherent *vs* spheres cell cultures.
(TIF)
