# Supplementary figures and images for: Spheres Derived from Lung Adenocarcinoma Pleural Effusions: Molecular Characterization and Tumor Engraftment
Source: PLoS One. 2011 Jul 18;6(7):e21320. doi: 10.1371/journal.pone.0021320 (PMC3138755; doi:10.1371/journal.pone.0021320)

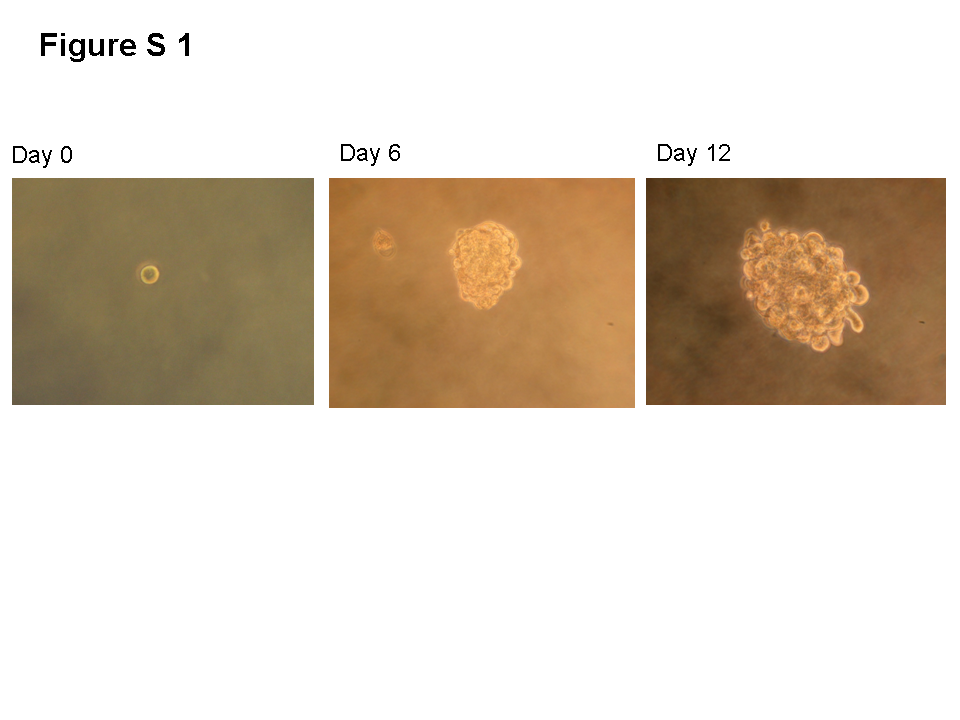

Supplement: Figure S1 — Sphere assays, self renewal ability of primary lung cancer cell. (TIF) [file pone.0021320.s001.tif]

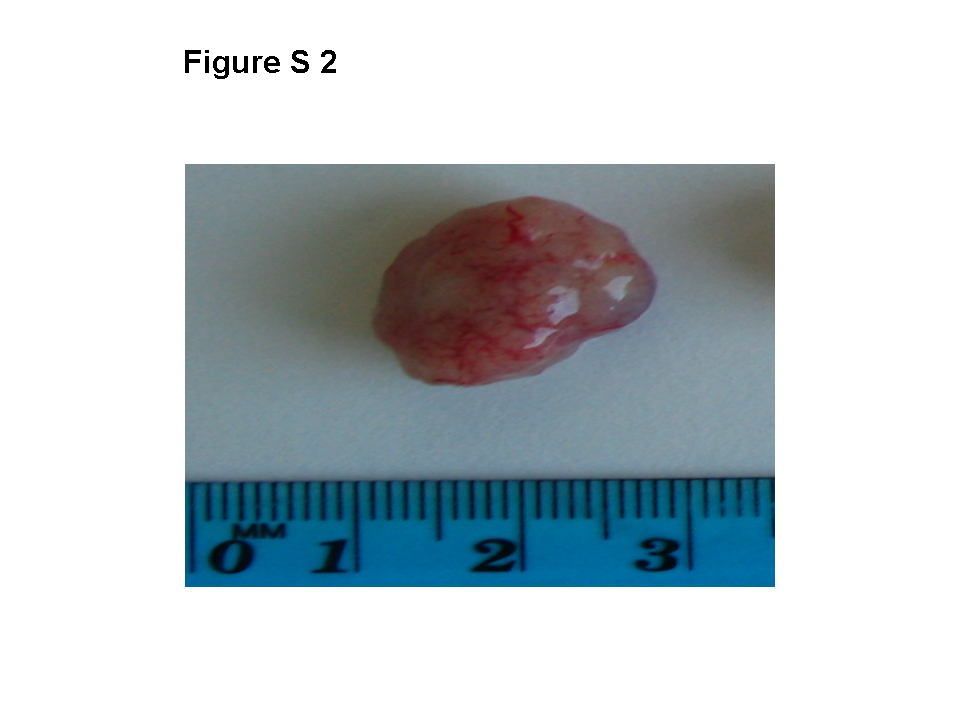

Supplement: Figure S2 — NOD/SCID mice subcutaneous tumor growth. (TIF) [file pone.0021320.s002.tif]

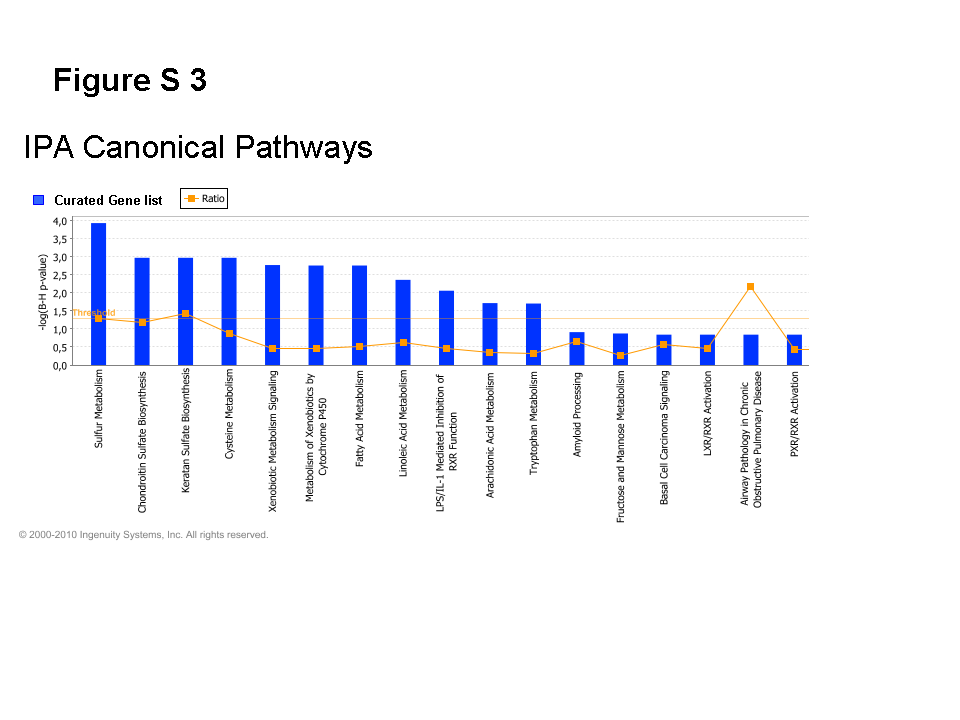

Supplement: Figure S3 — IPA Canonical Pathways. (TIF) [file pone.0021320.s003.tif]

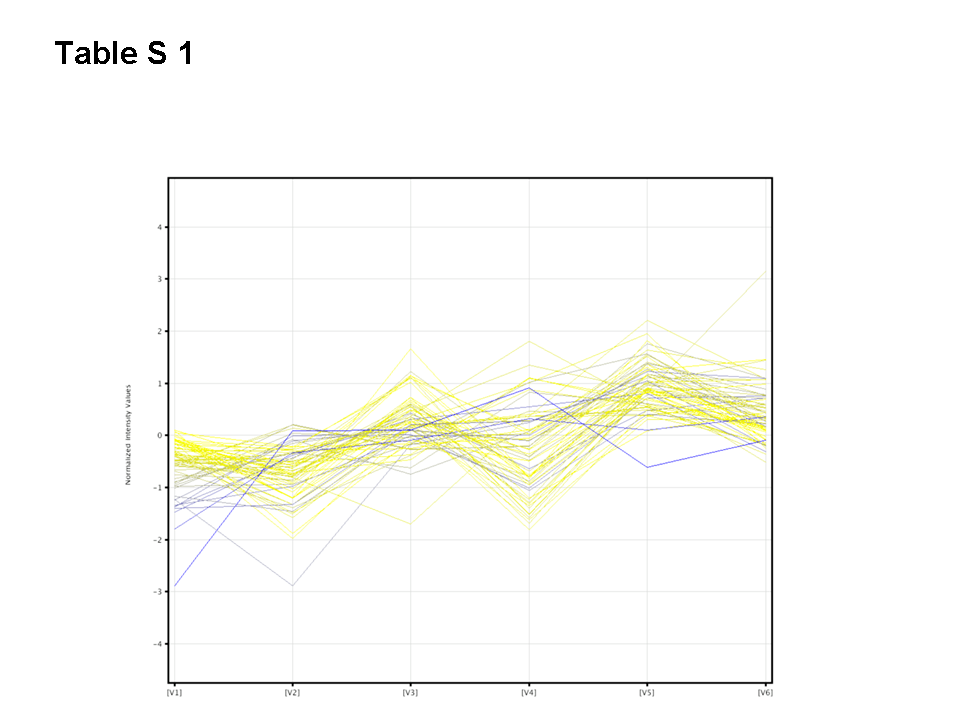

Supplement: Table S1 — Enriched GO terms and filtered by the Hypergeometric test and corrected using False Discovery Rate (FDR). (TIF) [file pone.0021320.s004.tif]

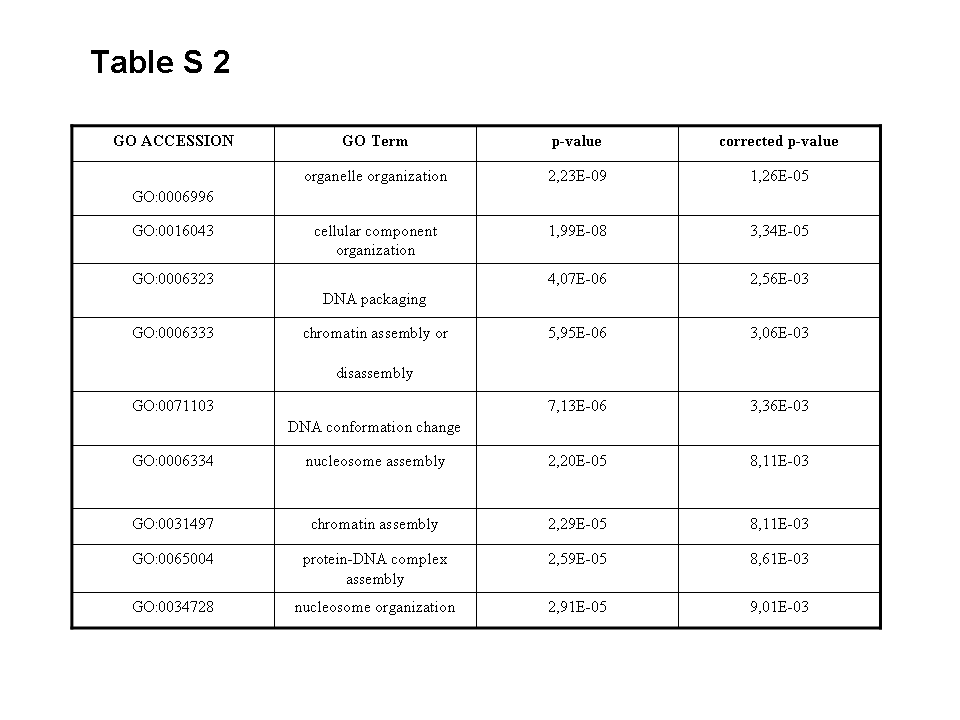

Supplement: Table S2 — Cluster, containing 69 probesets, was functionally annotated by Gene Ontology (GO) for Biological Process. (TIF) [file pone.0021320.s005.tif]

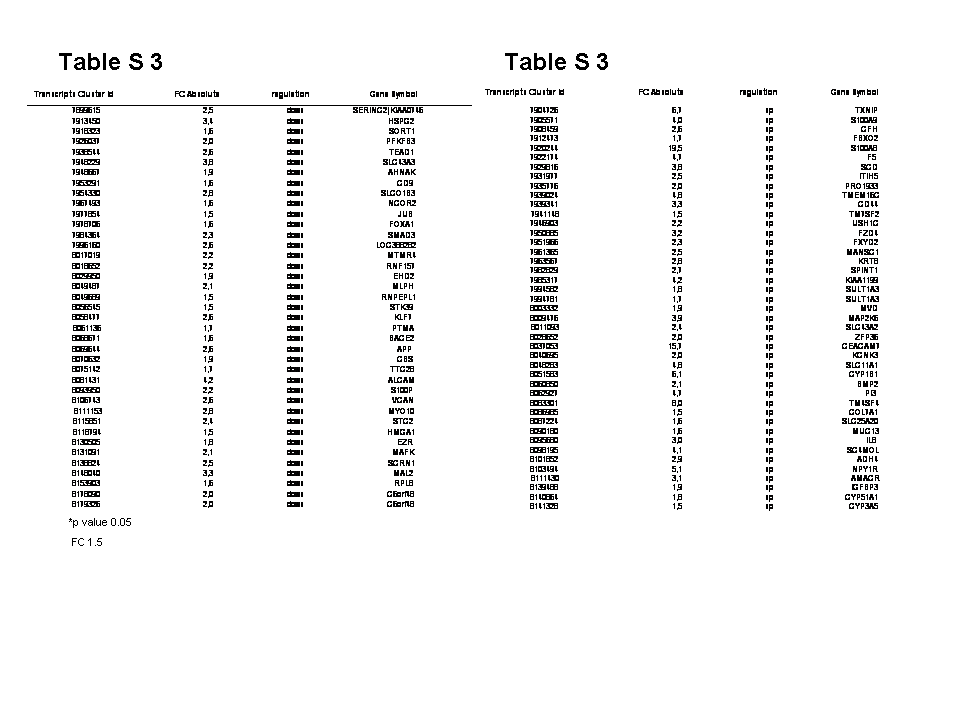

Supplement: Table S3 — 69 probesets expression profile over time. (TIF) [file pone.0021320.s006.tif]

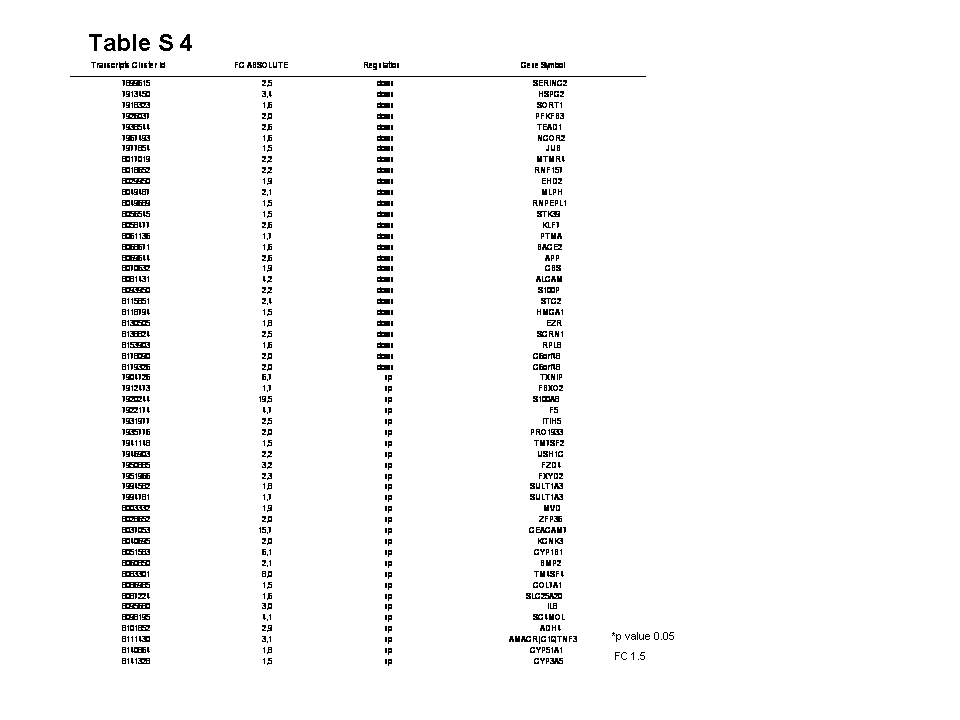

Supplement: Table S4 — Differential expressed probe sets of 82 genes in adherent vs spheres cell cultures. (TIF) [file pone.0021320.s007.tif]
